# Cholestatic Jaundice as a Paraneoplastic Manifestation of Prostate Cancer

**DOI:** 10.1155/2013/303727

**Published:** 2013-09-29

**Authors:** Tomomi Kuramoto, Hiroya Senzaki, Hiroyuki Koike, Kenji Yamagiwa, Shinobu Tamura, Tokuzou Fujimoto, Takeshi Inagaki

**Affiliations:** ^1^Department of Urology, Social Insurance Kinan Hospital, 46-70 Shinjou-chou, Tanabe-shi, Wakayama 646-8588, Japan; ^2^Department of Hematology/Oncology, Social Insurance Kinan Hospital, 46-70 Shinjou-chou, Tanabe-shi, Wakayama 646-8588, Japan; ^3^Department of Internal Medicine, Social Insurance Kinan Hospital, 46-70 Shinjou-chou, Tanabe-shi, Wakayama 646-8588, Japan

## Abstract

Paraneoplastic syndrome associated with prostate cancer is extremely rare. We report a patient who presented with cholestatic jaundice without biliary duct obstruction, hepatic involvement, or infection. After a few detailed examinations, prostate cancer was diagnosed. After treatment with bicalutamide and leuprolide, the patient's symptoms and laboratory abnormalities were reversed. Cholestatic jaundice was regarded as a paraneoplastic manifestation in this patient. The patient remains symptom-free after 14-month followup. Paraneoplastic syndrome should be considered in case of cholestatic jaundice without biliary duct obstruction, hepatic involvement, or infection.

## 1. Introduction

Cholestatic jaundice as the initial symptom in patients with metastatic prostate cancer is extremely rare. Hepatic metastasis or biliary duct obstruction are cited as the causes of cholestatic jaundice in the literature [1, 2].

More rarely, patients with advanced prostate cancer present with cholestatic jaundice without any evidence of biliary duct obstruction or hepatic infiltration. Cholestatic jaundice is attributed to the remote effects of the tumor, known as paraneoplastic syndrome, particularly in patients with nonmetastatic renal cell carcinoma (Stauffer's syndrome) [3]. 

However, prostate cancer presenting as cholestatic jaundice is extremely rare, and to our knowledge, only 4 cases of paraneoplastic cholestatic jaundice associated with prostate cancer have been reported in the literature [4–7]. 

We present a case of paraneoplastic cholestasis in prostate cancer and discuss the mechanism underlying the pathogenesis of this syndrome.

## 2. Case Report

A 75-year-old man was transported to our hospital by ambulance because of shock due to melena. Gastrointestinal endoscopy revealed a hemorrhagic gastric ulcer, for which endoscopic hemostasis was performed; clinical examination revealed jaundice. Head and neck examinations revealed several small, soft, painless, and movable left cervical lymph nodes. 

The liver and spleen were not palpable; however, a fist-sized mass was palpable around the umbilicus. Digital rectal examination revealed a walnut-sized prostate gland, and a stony hard mass was palpable behind the prostate.

**Figure 1:**
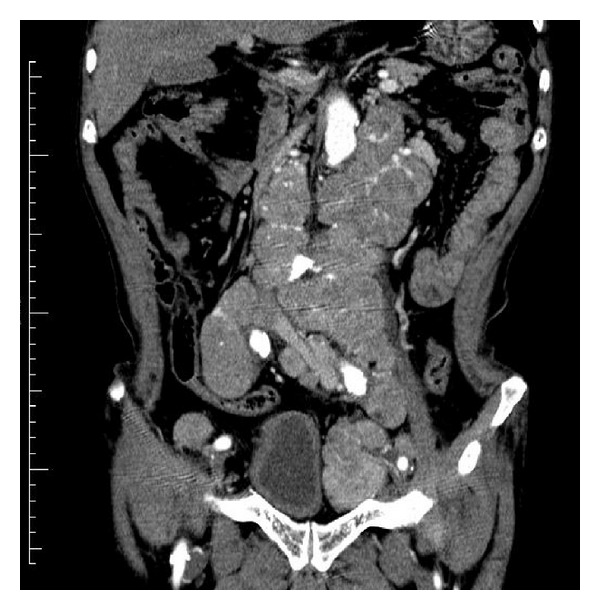
Abdominal computed tomography showing para-aortic and pelvic lymph node enlargement.

Laboratory analysis revealed the following: hemoglobin, 8.7 g/dL; white blood cell count 4700/*μ*L; thrombocyte count 16.9 × 10^4^/*μ*L; serum aspartate aminotransferase (AST) 45 U/L (normal range, 11–35 U/L); serum alanine aminotransferase (ALT) 42 U/L (normal range, 5–35 U/L); total bilirubin (t-bil) 17 mg/dL (normal range, 0.2–1.0 mg/dL); direct bilirubin (d-bil) 8.7 mg/dL (normal range, 0–0.4 mg/dL); and alkaline phosphatase (ALP) 44 U/L (normal range, 100–34 U/L). Urine analysis was positive for bilirubin but otherwise normal. Prostate-specific antigen (PSA) was 9862 ng/mL (normal range, 0–4 mg/mL). Abdominal computed tomography revealed para-aortic and pelvic lymph node enlargement but no abnormal findings in the liver, pancreas, spleen, or kidney (Figure 1). Magnetic resonance imaging revealed numerous enlarged lymph nodes in the pelvis. Bone scintigraphy revealed hot lesions on several ribs and the second lumbar vertebra. Prostate biopsy revealed adenocarcinoma (poorly differentiated, Gleason score 5 + 4). Antiandrogen treatment with bicalutamide (80 mg orally) was started, and after 14 days leuprolide was added (3.75 mg subcutaneously once a month) to the regimen. Twenty-eight days after the first subcutaneous injection of leuprolide, there was approximately 50% in the t-bil levels (from 17 to 10.5 mg/dL). After further 2.5 months, there levels returned to normal (Figure 3) following which AST, ALT, and ALP levels returned to normal 2 months later.

**Figure 2:**
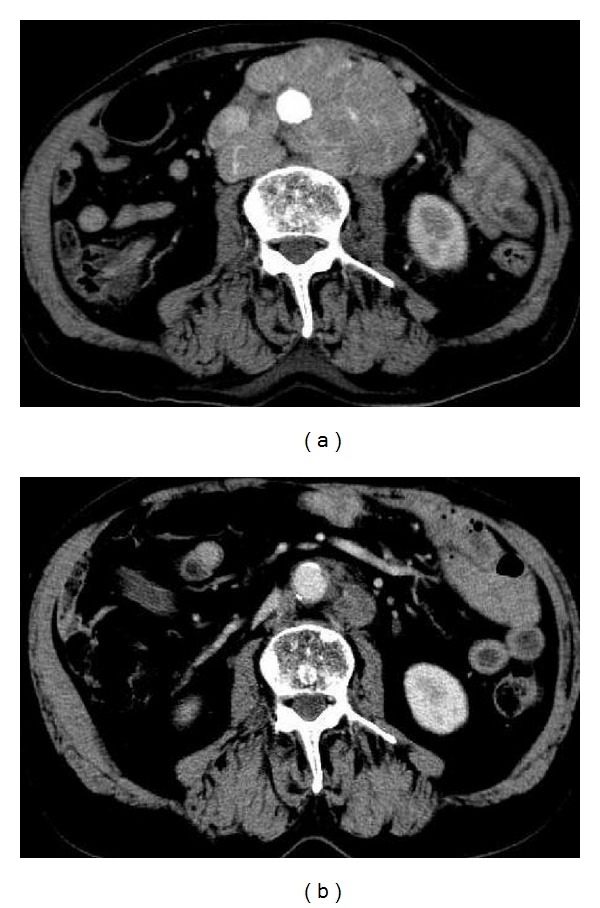
Abdomen computed tomography scans during treatment. (a) Prior to hormonal therapy, showing para-aortic and pelvic lymph node enlargement. (b) Six months after initiation of hormonal therapy, lymph node size decreased to more than 40%.

Hormonal therapy contributed to decrease in the size (by >40%) of para-aortic and pelvic lymph nodes (Figure 2). The patient achieved partial remission according to response evaluation criteria in solid tumors (RECIST) after a further 6 months.

**Figure 3:**
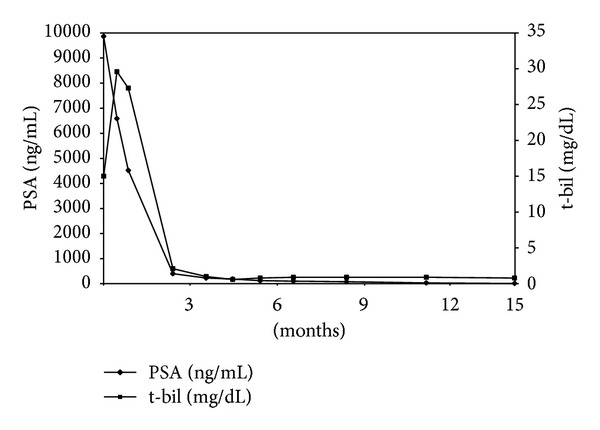
The patient's clinical course.

Furthermore, 1 year after initiation of hormonal therapy, PSA levels decreased from 9862 to 13 ng/mL (Figure 3); the patient is presently in remission and continues to receive leuprolide (3.75 mg) subcutaneously once a month and bicalutamide (80 mg) orally daily. 

## 3. Discussion

Paraneoplastic syndrome is defined as a group of symptoms that may develop when substances released by certain cancer cells disrupt the normal functions of surrounding cells and tissues. Prostate cancer is the second most common urological malignancy associated with paraneoplastic syndrome after renal cell carcinoma [8].

Hong et al. reported many types of paraneoplastic phenomena associated with prostate cancer. They classified paraneoplastic syndrome into endocrine, hematological, dermatological, neurological, inflammatory, and other types according to clinical symptoms [9]. Among these, paraneoplastic cholestasis was uncommon, and the pathogenesis of paraneoplastic syndrome remains unknown. Blay et al. reported that interleukin-6 (IL-6)is involved in the physiopathology of paraneoplastic cholestasis in renal cell carcinoma (Stauffer's syndrome) [10].

In our case, serum IL-6 levels decreased from 8.4 pg/mL (normal range, <4 pg/mL) before treatment to 2.8 pg/mL 4 months after treatment.

The literature includes 4 case reports cholestasis as a paraneoplastic manifestation associated with prostate cancer without metastatic hepatic infiltration, biliary duct obstruction, or evidence of an infections etiology [4–7]. 

In our case, the patient had bone metastasis and enlarged lymph nodes at the time of diagnosis, and no biliary duct obstruction was observed because of lymph node involvement. The patient remains symptom-free and without biochemical relapse 1 year after initiation of antiandrogen treatment. 

Because the jaundice evident at the start of treatment disappeared after effective hormonal treatment, we consider that this symptom was a paraneoplastic manifestation of the underlying prostate cancer. 

In conclusion, paraneoplastic cholestasis should be considered in the absence of metastatic infiltration of the liver, metastatic extrahepatic biliary duct obstruction, or an infectious etiology in prostate cancer.
